# A review of plant phenolics and endozoochory

**DOI:** 10.1002/ece3.70255

**Published:** 2024-09-16

**Authors:** Samuel A. Krebs, Michael L. Schummer

**Affiliations:** ^1^ Department of Environmental Biology State University of New York College of Environmental Science and Forestry (SUNY ESF) Syracuse New York USA

**Keywords:** bird vision, coevolution, phenolics, plant defense, seed dispersal, UV reflection

## Abstract

Phenolic compounds (phenolics) are secondary metabolites ubiquitous across plants. The earliest phenolics are linked to plants' successful transition from an aquatic to a terrestrial environment, serving as protection against damaging ultraviolet (UV) radiation, and as antioxidants to reduce oxidative stress in an atmosphere with an increasingly high O_2_:CO_2_ ratio. In modern plants, phenolics are best known for the defense against fungal and bacterial pathogens and as antifeedants that deter herbivory. Phenolics also play a role in seed dormancy, delaying germination, and lengthening viability in the seed bank. Many plants' seeds are endozoochorous – dispersed by animals, like birds, who eat and later excrete the seeds. Plants send visual signals to attract birds with UV‐sensitive (UVS) vision for pollination and seed dispersal. As fruits ripen, antioxidant activity and phenolic content decrease. The waxy cuticle of fruits increases in UV reflection as phenolic rings, which absorb UV light, degrade. The UV contrast that birds detect may act as an honest signal, indicating nutritional changes in the fruit. However, there is little evidence to support the evolution of UV coloration during ripening being driven by frugivore selection. Antioxidant properties of fruit phenolics may be dually adaptive in plants and avian frugivores.

## Graphical abstract

Phenolic compounds, vital secondary metabolites in plants, evolved to protect against UV radiation and oxidative stress during their transition to terrestrial environments. In modern plants, phenolics serve primarily as defense mechanisms against pathogens and herbivores, while also influencing seed dormancy and viability. As fruits ripen, a decrease in phenolic content alters UV reflectance, potentially signaling nutritional changes to birds, though evidence for frugivore‐driven evolution remains limited.

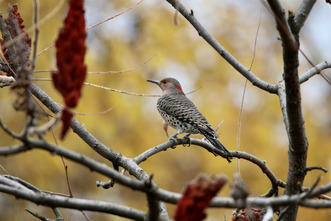

## 1 INTRODUCTION

This paper underscores the ecological and evolutionary importance of phenolic compounds. By exploring their multifaceted roles in plants, this research highlights how phenolic compounds have influenced adaptation to a terrestrial environment, defensive strategies against herbivory and pathogens, and reproductive success including seed dispersal and germination, contributing to the resilience, and adaptability of plant species. This study examines the interaction between plants and their animal dispersers. Focusing on endozoochory, we explore how phenolics factor in by attracting dispersers and the effects of animal digestion on seed germination. We investigate the evolutionary and ecological relationship between today's predominant frugivores, birds, and fruit signaling strategies which may have coevolved with the ability of birds to see in the ultraviolet spectrum.

## 2 AN OVERVIEW OF PHENOLICS

Phenolic compounds, hereafter phenolics, are one of the three major classes of secondary metabolites which are chemical compounds found in plants that are not directly involved in primary metabolism (e.g., photosynthesis, respiration, etc.) (Taiz & Zeiger, 2006). Phenolics are ubiquitous within the plant kingdom but uncommon in bacteria, fungi, and algae. The nearly 10,000 different phenolics are united by the presence of an aromatic ring with one (phenol) or more (polyphenol) hydroxyl functional groups (Vuolo et al., 2019). Plant phenolics naturally arise from one of two pathways: the shikimate/phenylpropanoid pathway or the “polyketide” acetate/malonate pathway (Lattanzio et al., 2009). Phenolics can be divided at the molecular level into subgroups based on the construction of their carbon skeleton, kind of substituent, and degree of polymerization (Waterman & Mole, 1994). Subgroups include the simple phenolic compounds (simple phenolics and phenolic acids), the polyphenols (flavonoids and tannins), and others (stilbenes, lignans, and lignins) (Al Mamari, 2021). The flavonoids are further divided into anthocyanins, flavones, flavonols, and isoflavones (Table 1; Taiz & Zeiger, 2006).

**TABLE 1:** Phenolic compounds in seeds.

Classes		Example of abundant seed phenolic compounds	Functions and effects in seeds	References
Coumarins		Coumarin, 1,2‐benzopyrone, fraxetin, isofraxidin	Antifeedant, pathogen defense, germination inhibitor	Abenavoli et al. (2006), Brooker et al. (2008), Matos et al. (2015), Mustafa et al. (2018), and Yin et al. (2014)
Stilbenes		Resveratrol, transresveratrol, piceid, viniferin, pterostilbene	Antioxidant, pathogen defense	Counet et al. (2006), Hasan & Bae (2017), and Nayak et al. (2019)
Flavanoids	Flavanols	Quercetin, quercetin‐3‐O‐rhamnoside, kaempferol‐3‐O‐rhamnoside, myricetin	Antioxidant, embryo protection, seed dormancy, pathogen defense	Debeaujon et al. (2000), Figueroa et al. (2018), Lepiniec et al. (2006), MacGregor et al. (2015), and Singh et al. (2017)
	Anthocyanins	Cyanidin, delphinidin, malvidin, peonidin, pelargonidin	Antioxidant, pigmentation, pathogen defense	Shirley (1998) and Singh et al. (2017)
	Isoflavones	Daidzein, genistein, glycitein, sissostrin	Antioxidant, pathogen defense	Corso et al. (2020), Lee et al. (2017), Shirley (1998), Singh et al. (2017), and Xu et al. (2019)
	Flavones	Apigenin, luteolin, isoorientin, diosmetin	Antioxidant	Corso et al. (2020), Figueroa et al. (2018), and Singh et al. (2017)
	Flavanones	Naringenin, sakuranetin, hesperitin, chalcone, eriodictyol	Antioxidant	Figueroa et al. (2018), Guerrero‐Castillo et al. (2019), and Singh et al. (2017)
	Flavan‐3‐ols	Catechin, epicatechin, epigallocatechin, gallocatechin, proanthocyanidins, procyanidins, prodelphinidins, procyanidin dimers/trimers/pentamers	Pigmentation, germination inhibitor, seed dormancy, pathogen defense, antifeedant, antioxidant, embryo protection	Corso et al. (2020), Figueroa et al. (2018), Shirley (1998), and Singh et al. (2017)
	Flavan‐4‐ols	Phlobaphenes	Antioxidant, seed maturation, pathogen defense	Lepiniec et al. (2006) and Shirley (1998)

*Note*: Their classification, examples of representative compounds, and their functions and effects.

The phenological and spatial patterns of phenolics are complex; in a single species, it can be different between developmental stages, individuals, or populations. To properly function, phenolics accumulate in specific tissues or cell types where subcellular localization is highly regulated (Lattanzio et al., 2009). Phenolics are often transported from source cells to other nearby cells and sometimes sent to distant tissues or organs. There is significant compartmentalization of phenolics with accumulation occurring in the vacuoles of spongy cells (Santiago et al., 2000), covalently linked to the cell walls (Iiyama et al., 1994), in waxes (Schmutz et al., 1993), and other external surfaces (Cuadra & Harborne, 1996).

## 3 THE EARLY ROLE OF PHENOLICS IN PLANTS

Plants and their phenolics evolved together. *Charophyceae* and *Chlorophyta*, green algae likely to be the ancestor of land plants, possess sporopollenin which is linked to acetolysis‐resistant phenolics in modern angiosperms (Stafford, 2000). Bryophytes, often considered the first land plants, have flavonoids, flavones, and lignans. Flavonoids, water‐soluble polyphenols with 15 carbon atoms, occur in approximately 60% of mosses (*Bryophyta*) and liverworts (*Marchantiophyta*) (Markham, 1990). Flavones, a subgroup of flavonoids, are widely distributed among mosses and liverworts (Markham et al., 1998). The same lignans found in liverworts and hornworts (*Anthocerophyta*) are present in modern vascular plants along with a broad range of phenolics with differences among woody and herbaceous plants (Stafford, 2000).

The earliest phenolics are strongly linked to the transition from an aquatic to a terrestrial environment (Delaux et al., 2012). Living on land meant increased exposure to harmful UV and visible radiation (Cheynier et al., 2013). Just like sporopollenin in green algae that protects against UV radiation (Xiong et al., 1997), simple phenolics and flavonoids in early land plants may have been crucial as UV‐B (280–320 nm) absorbing filters (Waterman & Mole, 1994). Another result of moving to land was exposure to an atmosphere with an increasingly high O_2_:CO_2_ ratio. Thus, plants needed a method of reducing oxidative stress. As a result, aerobic metabolic processes produced more reactive oxygen species (ROS) (Apel & Hirt, 2004; Igamberdiev & Lea, 2006). In tandem, an increase in the diversity and complexity of secondary metabolites (Graham et al., 2004) led to phenolics with strong antioxidant properties (Cooper‐Driver & Bhattacharya, 1998) able to detoxify harmful ROS and alleviate oxidative stress (Chaki et al., 2020; Sakihama et al., 2002). It is the multifunctionality of phenolics that has made them influential in the success of modern plants (Table 1; Karabourniotis et al., 2014).

## 4 PHENOLICS AS HERBIVORE DETERRENTS

Perhaps the most well‐known function of plant phenolics is for defense against herbivores and pathogens. Unlike animals, plants are sessile and cannot escape from biotic and abiotic stressors. Being linked to the ground means they must create metabolic products to survive attackers because escape is not an option (Taiz & Zeiger, 2006). Allocating resources to secondary metabolism is antagonistic to primary metabolism – a choice of grow or defend (Herms & Mattson, 1992). This means investing photosynthetic products into growth should be mutually exclusive of defense (Karabourniotis et al., 2014) and explains the negative correlation between a plant's photosynthetic ability and phenolic concentration (Sumbele et al., 2012). Balance is determined by an environment's unique combination of competition and herbivory that favors carbon allocation towards levels of growth and defense (Herms & Mattson, 1992). The fitness costs of defense may be more complex than the diversion of limited resources from growth and reproduction. A meta‐analysis by Koricheva (2002) found that the interactions between plants and their environment (e.g., competitors, pollinators, different types of herbivores, abiotic stresses, and natural enemies) may play a significant role in the fitness cost of defenses. Despite the trade‐off of investing in defense, phylogenetic patterns indicate the evolutionary trend in angiosperms towards increasingly toxic phenolics (Harborne, 1990). Over time, as plants have diverged they have acquired a greater number of defensive compounds belonging to more diverse chemical pathways, making it harder for herbivores to adapt to them (Becerra et al., 2009).

There is a wide array of defensive capabilities among phenolic subgroups (Taiz & Zeiger, 2006). Phenolics are produced as part of normal plant development and in response to physical damage (Pratyusha, 2022). Due to its physical rigidity and chemical durability, lignin deters most animals by making the plant indigestible (Jung & Allen, 1995). Lignification is a common response to microbial disease and can prevent pathogens from spreading (Vance et al., 1980). Phenolic acids and flavonoids that function as antioxidants in a healthy plant can become harmful prooxidants to insects by causing oxidative damage to their tissues (Grace, 2005). Phytoalexins are antimicrobial isoflavones produced after bacterial or fungal infection that likely defend against pathogen development (Hammerschmidt, 1999; Keen, 1981). Tannins are antifeedants that repel herbivory due to their general toxicity, decreasing protein digestion in vertebrates and increasing the production of ROS in insects (Barbehenn & Constabel, 2011). The outer cell layers of fruits often have high tannin concentrations, deterring herbivory until concentrations decrease with enzyme activity during ripening (Goldstein & Swain, 1963; Zarei et al., 2011). Tannins also defend against fungal and bacterial decay, with localization in nonliving heartwood (Scalbert, 1992).

Empirical studies provide insight into the antifeedant role of phenolics. Herbivores can select food sources based on the genetic variation in patterns of phenolics (Herms & Mattson, 1992). Potato cultivars with higher levels of phenolics have been found to be less attractive to invertebrate pests (Bennett & Wallsgrove, 1994). Similarly, bird pests are highly selective and avoid sorghum cultivars with high tannin levels compared to those without tannins (Butler, 1989). Chemical assays of crop tannin levels can be useful for farmers in predicting bird resistance (Hoshino & Duncan, 1982; McMillian et al., 1972; Tipton et al., 1970). In trees such as quaking aspen (*Populus tremuloides*), willow (*Salix* spp.), and balsam poplar (*Populus balsamifera*), younger shoots tend to have higher levels of phenolics (Lindroth & Hwang, 1996). Studies show snowshoe hares (*Lepus americanus*) and mountain hares (*Lepus timidus*) preferentially consume older shoots with the lower phenolic content and avoid relatively unpalatable juvenile shoots (Bryant, 1981; Reichardt et al., 1990; Tahvanainen et al., 1985). However, antifeedant capability is likely chemical‐specific and not related to the total quantity of various phenolics present. Ruffed grouse (*Bonasa umbellus*) diets rely upon aspen buds and catkins during winter and spring. These herbivores have adapted to avoid antifeedant phenolics. Grouse selection of buds and catkins is towards those with lower levels of coniferyl benzoate, an ester formed by condensation of coniferol and benzoic acid (Jakubas & Gullion, 1991).

## 5 REGULATING SEED GERMINATION

Each seed contains an inactive embryo and germination is the beginning of its growth (Toole et al., 1956). During germination, respiration rapidly increases and involves several enzymatic pathways: glycolysis, the oxidative pentose phosphate pathway (OPPP), the tricarboxylic cycle, and oxidative phosphorylation (Botha et al., 1992). Dormancy is the period before viable temperate seeds germinate. Seeds can be prevented from germinating because of a physical barrier (coat‐enhanced dormancy), because of the biochemical conditions within the embryo itself (embryo dormancy), or a combination of both (double dormancy) (Baskin & Baskin, 2021; Bewley, 1997). Dormancy serves an important role in protection from unsuitable conditions too harsh for seedling survival (Koornneef et al., 2002). Length of dormancy and timing germination is important among years as well as growing seasons because of high variability in stressors, such as competition and precipitation (Ten Brink et al., 2020).

Seed coats and embryos contain naturally occurring phenolics that affect dormancy (Lattanzio et al., 2009). Phenolics delay germination by suppressing enzymes involved in glycolysis (ALD and PGI) and OPPP (G6P‐DH), disrupting cellular respiration (Gharachorloo et al., 2013; Muscolo et al., 2001). Phenolics also inhibit amino acid transport and protein synthesis (Van Sumere, 1972). Total phenolics increase after seed dispersal and degrade with time, suggesting an evolutionary mechanism that lengthens viability in the seed bank (Inácio et al., 2013). In addition to length of time, temperature affects phenolic degradation. Colder temperatures significantly decrease phenolic content and help explain why cold stratification is effective in germinating seeds (Willemsen & Rice, 1972).

In allelopathic plants, phenolics inhibit the germination of other competing plant's seeds (Williams & Hoagland, 1982). Phenolics leach into soil and inhibit seed germination by as much as 16–61% depending on which compounds and their concentrations (Muscolo et al., 2001). Additionally, these allelopathic chemicals can affect already established plants by modifying chloroplast and mitochondria membranes, preventing intracellular transport, and inhibiting the hydrolysis of ATP (Moreland & Novitzky, 1987). Not only are photosynthesis and respiration affected but cell membrane permeability, cell division, protein synthesis, and hormone activity are each affected by phenolic allelochemicals (Li et al., 2010).

## 6 ENDOZOOCHORY AND SEED GERMINATION

In nature, embryo dormancy is often overcome by exposure to cold temperatures during winter (summer annuals) or warm temperatures during summer (winter annuals) (Baskin & Baskin, 2004). Seed‐coat dormancy can be broken, or scarified, either mechanically or chemically (Mousavi et al., 2011). Freeze–thaw cycles can physically break the seed coat (Kimura & Islam, 2012). Microbial activity can chemically scarify seed coats and enhance germination (Morpeth & Hall, 2000). Animals can provide physical and chemical scarification while acting as a seed transportation vessel.

Many extant plants (64% of gymnosperms and 27% of angiosperms) produce seeds that attract animal dispersers (Herrera & Pellmyr, 2009). Animal dispersal is particularly important in naturally fragmented habitats such as wetlands (Charalambidou et al., 2003). Understanding animal movement and their contribution to seed dispersal is increasingly important with anthropogenic pressures hindering plant dispersal (Tucker et al., 2021). Zoochory is the general term to describe any mode of animal dispersal. Epizoochory is dispersal by outside of the body, such as burrs stuck on fur (Iluz, 2011). Synzoochory is dispersal by seed‐caching animals that do not consume the seed, such as squirrels (*Sciurus* spp.) burying acorns. Endozoochory is the purposeful ingestion of seeds, often involving a fleshy fruit surrounding the seed to entice the consumer. Endozoochory is the most widespread animal dispersal method of extant plants and can be considered the most genuine (Herrera, 1989). Endozoochory provides a means of scarification for seeds that are relatively small or have hard enough coats to withstand passage through digestive tracts (Krefting & Roe, 1949).

It has been long debated how effective animal guts are at scarifying dormant seeds. Early studies found that germination could benefit from passage through animal digestive tracts but was dependent on species of seed and animal consumers (Krefting & Roe, 1949; Swank, 1944). The notion that effectiveness is dependent on the animal consumer is supported by a variety of studies (Table 2). *Prosopis flexuosa* seeds consumed by herbivorous mammals (*Dolichotis patagonum* and *Lama guanicoe*) and tortoises (*Chelnoidis chilensis*) experienced significantly increased germinability whereas pampas fox (*Lycalopex gymnocercus*) and greater rhea (*Rhea americana*) ingestion had no noticeable effect (Campos et al., 2020). Black mulberry (*Morus nigra*), black bryony (*Tamus communis*), and red‐berry mistletoe (*Viscum cruciatum*) seeds ingested by common blackbirds (*Turdus merula*) had significantly greater germination percentages than those eaten by white‐spectacled bulbuls (*Pycnonotus xanthopygos*) (Barnea et al., 1991). Traveset and Willson (1997) found little variation in multiple seed species' germination rates after consumption by bird and bear digestive systems – only one plant, red elderberry (*Sambucus racemosa*), appeared to benefit more from the guts of varied thrush (*Ixoreus naevius*) than other animals. Some seeds experience more prompt germination at the expense of reduced viability. For example, cattle and rodent endozoochory enhance legume seed germination but decreases overall viability (Campos & Ojeda, 1997). It is important to note that germination may be less successful after animal consumption, furthering the notion that germinability is highly dependent on the plant and animal species involved (Charalambidou et al., 2005; Cosyns et al., 2005).

**TABLE 2:** Seeds tested with frugivore bird species/family, effect(s) of ingestion on seed germination rate (+, enhancement; —, inhibition; 0, neutral), fruit type (F, fleshy; D, dry), and seed size (S, small, <5 mm; M, medium, 5–10 mm; L, large, >10 mm).

Plant species (Family)	Disperser species	Disperser family	Effect(s)	Fruit type	Seed size	References
Anacardiaceae						
*Rhus glabra*	*Colinus virginianus*	Odontophoridae	+	F	S	Krefting and Roe (1949)
*Toxicodendron vernix*	*Colinus virginianus*	Odontophoridae	+	F	S	Krefting and Roe (1949)
*Toxicodendron vernix*	*Phasianus colchicus ssp. torquatus*	Phasianidae	+	F	S	Krefting and Roe (1949)
Apocynaceae						
*Arum hygrophilum*	*Turdus merula*	Turdidae	0—	F	S	Barnea et al. (1991)
*Oplopanax horridus*	*Ixoreus naevius*	Turdidae	00	F	M	Traveset and Willson (1997)
*Oplopanax horridus*	*Turdus migratorius*	Turdidae	00	F	M	Traveset and Willson (1997)
Caprifoliaceae						
*Lonicera tatarica*	*Dumetella carolinensis*	Mimidae	0	F	S	Krefting and Roe (1949)
*Lonicera tatarica*	*Turdus migratorius*	Turdidae	+	F	S	Krefting and Roe (1949)
*Sambucus canadensis*	*Dumetella carolinensis*	Mimidae	+	F	S	Krefting and Roe (1949)
*Sambucus canadensis*	*Turdus migratorius*	Turdidae	+	F	S	Krefting and Roe (1949)
*Sambucus canadensis*	*Phasianus colchicus* ssp*. torquatus*	Phasianidae	−	F	S	Krefting and Roe (1949)
*Sambucus racemosa*	*Ixoreus naevius*	Turdidae	0+	F	S	Traveset and Willson (1997)
*Sambucus racemosa*	*Turdus migratorius*	Turdidae	0+	F	S	Traveset and Willson (1997)
Cornaceae						
*Cornus racemosa*	*Colinus virginianus*		−	F	S	Krefting and Roe (1949)
*Cornus racemosa*	*Phasianus colchicus* ssp. *torquatus*	Phasianidae	−	F	S	Krefting and Roe (1949)
*Cornus stolonifera*	*Colinus virginianus*	Odontophoridae	−	F	S	Krefting and Roe (1949)
*Cornus stolonifera*	*Phasianus colchicus* ssp. *torquatus*	Phasianidae	−	F	S	Krefting and Roe (1949)
Ephedraceae						
*Ephedra campylopoda*	*Turdus merula*	Turdidae	00	F	S	Barnea et al. (1991)
*Ephedra campylopoda*	*Pycnonotus xanthopygos*	Pycnonotidae	00	F	S	Barnea et al. (1991)
Ericaceae						
*Vaccinum alaskaense, V. ovalifolium*	*Ixoreus naevius*	Turdidae	00	F	S	Traveset and Willson (1997)
*Vaccinum alaskaense, V. ovalifolium*	*Turdus migratorius*	Turdidae	00	F	S	Traveset and Willson (1997)
Liliaceae						
*Asparagus aphyllus*	*Erithacus rubecula*	Muscicapidae	00	F	S	Barnea et al. (1991)
*Asparagus aphyllus*	*Turdus merula*	Turdidae	00	F	S	Barnea et al. (1991)
*Streptopus amplexifolius*	*Ixoreus naevius*	Turdidae	00	F	S	Traveset and Willson (1997)
*Streptopus amplexifolius*	*Turdus migratorius*	Turdidae	00	F	S	Traveset and Willson (1997)
Moraceae						
*Morus alba*	*Turdus migratorius*	Turdidae	+	F	S	Krefting and Roe (1949)
*Morus nigra*	*Turdus merula*	Turdidae	++	F	S	Barnea et al. (1991)
*Morus nigra*	*Pycnonotus xanthopygos*	Pycnonotidae	++	F	S	Barnea et al. (1991)
Myrtaceae						
*Myrtus communis*	*Turdus merula*	Turdidae	0+	F	S	Barnea et al. (1991)
*Myrtus communis*	*Pycnonotus xanthopygos*	Pycnonotidae	0+	F	S	Barnea et al. (1991)
Rhamnaceae						
*Rhamnus alaternus*	*Turdus merula*	Turdidae	++	F	S	Barnea et al. (1991)
*Rhamnus alaternus*	*Pycnonotus xanthopygos*	Pycnonotidae	++	F	S	Barnea et al. (1991)
*Rhamnus palaestinus*	*Turdus merula*	Turdidae	+—	F	S	Barnea et al. (1991)
*Rhamnus palaestinus*	*Pycnonotus xanthopygos*	Pycnonotidae	+0	F	S	Barnea et al. (1991)
Rosaceae						
*Prunus serotina*	*Turdus migratorius*	Turdidae	+	F	M	Krefting and Roe (1949)
*Prunus virginiana*	*Phasianus colchicus*	Phasianidae	−	F	M	Krefting and Roe (1949)
*Rosa blanda*	*Colinus virginianus*	Odontophoridae	−	F	S	Krefting and Roe (1949)
*Rosa blanda*	*Phasianus colchicus*	Phasianidae	−	F	S	Krefting and Roe (1949)
*Rosa* sp.	*Tympanuchus phasianellus*	Phasianidae	−	F	S	Krefting and Roe (1949)
*Rosa* sp.	*Phasianus colchicus*	Phasianidae	+	F	S	Krefting and Roe (1949)
*Rubus occidentalis*	*Bombycilla cedrorum*	Bombycillidae	−	F	S	Krefting and Roe (1949)
*Rubus sanctus*	*Turdus merula*	Turdidae	—0	F	S	Barnea et al. (1991)
*Rubus sanctus*	*Pycnonotus xanthopygos*	Pycnonotidae	—0	F	S	Barnea et al. (1991)
*Rubus* sp.	*Bombycilla cedrorum*	Bombycillidae	0	F	S	Krefting and Roe (1949)
*Rubus* sp.	*Dumetella carolinensis*	Mimidae	+	F	S	Krefting and Roe (1949)
*Rubus* sp.	*Turdus migratorius*	Turdidae	+	F	S	Krefting and Roe (1949)
*Rubus spectabilis*	*Ixoreus naevius*	Turdidae	0+	F	S	Traveset and Willson (1997)
Rubiaceae						
*Rubia tenuifolia*	*Turdus merula*	Turdidae	0+	F	S	Barnea et al. (1991)
*Rubia tenuifolia*	*Pycnonotus xanthopygos*	Pycnonotidae	0+	F	S	Barnea et al. (1991)
Saxifragaceae						
*Ribes bracteosum*	*Ixoreus naevius*	Turdidae	00	F	S	Traveset and Willson (1997)
*Ribes bracteosum*	*Turdus migratorius*	Turdidae	00	F	S	Traveset and Willson (1997)
*Ribes missouriense*	*Bombycilla cedrorum*	Bombycillidae	+	F	S	Krefting and Roe (1949)
*Ribes missouriense*	*Dumetella carolinensis*	Mimidae	+	F	S	Krefting and Roe (1949)
Vitaceae						
*Vitis riparia*	*Colinus virginianus*	Odontophoridae	−	F	S	Krefting and Roe (1949)
*Vitis riparia*	*Phasianus colchicus*	Phasianidae	−	F	S	Krefting and Roe (1949)

It appears alien animals may decrease the germinability of seeds more than their native counterparts. In Argentina, invasive European wild boars (*Sus scrofa*) decrease the germinability of all seeds they consume (Campos & Ojeda, 1997). In the Galápagos Islands, native giant tortoises (*Chelonoidis niger*) significantly improve the speed and percentage of germination of native tomato seeds while no seeds germinated after consumption by invasive rats (Rick & Bowman, 1961). In the Canary Islands, the viability of *Rubia fruticosa* seeds is much greater after ingestion by native reptiles and birds than by exotic mammals like European rabbits (*Oryctolagus cuniculus*) and Barbary ground squirrels (*Atlantoxerus getulus*) (Nogales et al., 2005).

There is also debate over the effect of retention time, or how long it takes a seed to go through the digestive tract. Barnea et al. (1991) found seeds with longer retention times had increased seed coat permeability and therefore more successful germination. Likewise, *Capparis spinosa* seeds with longer retention times in lizard digestive tracts broke dormancy and increased germination rate (Yang et al., 2021). Alternatively, wigeon grass (*Ruppia maritima*) seeds ingested by five dabbling duck species were more likely to germinate if the retention time was shorter (Charalambidou et al., 2003). Campos et al. (2020) found that retention time was not the most important factor because, among five consumers, the species with a significantly longer retention time (*Chelonoidis chilensis*) did not have the lowest seed viability or germinability. Traveset and Willson (1997) also found retention time was unimportant for Alaskan native shrub and herb seeds.

It is more likely that the nature of physical and chemical treatment the seed experiences in the gut is more important than the time it takes to pass through (Schupp, 1993). A recent study by van Leeuwen et al. (2023) on waterfowl digestion found that species‐specific viability was entirely dependent on the birds' gizzard, where foods are mechanically digested, whereas germinability was dependent on intestinal digestion. An extensive literature review of 366 plant species from 76 publications by Soltani et al. (2018) emphasizes the significance of the type of dormancy. Germination of all physically dormant seeds (coat‐enhanced dormancy) improved after endozoochory regardless of size. This suggests animal digestion may decrease the seed coat phenolic content that would typically suppress germination.

## 7 AVIAN VISION: FORAGING USING ULTRAVIOLET SIGNALS

Birds, with few exceptions, rely on their ability to visually assess their surroundings more than any other terrestrial vertebrate (Jones et al., 2007). There are only a handful of extant birds where vision is not the primary sense, such as kiwis (*Apteryx* spp.) whose vision likely regressed in the absence of predation (Martin, 2007). High visual awareness helps birds to navigate surroundings, avoid predators, and assess potential mates. Their acute vision, particularly sensitivity in the near ultraviolet, is useful in foraging (Hart, 2001). For example, the first bird known to use UV visual cues for foraging was the common kestrel (*Falco tinnunculus*) which detects UV‐reflecting scent trails of voles (Viitala et al., 1995). Although birds are sensitive to a broader range of light, their sensitivity to contrast between wavelengths is much less than that of humans. This means that there needs to be higher degrees of contrast for birds to detect detail while foraging for food (Martin, 2022).

The avian retina is generally more advanced and complex than that of humans (Husband & Shimizu, 2001). A bird's interpretation of color is different from ours and can differentiate between more wavelengths, giving them significantly greater spectral resolution (Martin, 2022). For most, this resolution is across a broad spectrum from ultraviolet (UV) to far‐red light (Cuthill et al., 2000). UV vision is a general property of birds and is only absent in relatively few species (Goldsmith, 1990). This extended range is a result of colored oil droplets and an additional cone type for a total of 4 (tetrachromacy) compared to our 3 (Vorobyev et al., 1998). The few who have evolved towards decreased UV sensitivity may owe this difference to additional pigmentation in the eye lens, likely protecting against UV damage to the retina (Olsson et al., 2021).

Bird species sensitive to near UV light (300–400 nm) can be categorized into two classes based on the maximal sensitivity of their short‐wave type 1 (SWS1) cones (Burns & Shultz, 2012). Species that have SWS1 cones with pigments with maximal sensitivity between 355 and 370 nm are classified as UV‐sensitive (UVS) whereas those with maximal sensitivity between 405 and 420 nm are violet‐sensitive (VS) species (Cuthill, 2006; Wilkie et al., 2000). Whether a species has UVS or VS vision is caused by a single amino acid substitution and is simple to determine through genomic sequencing of the SWS1 cone opsin gene (Ödeen & Håstad, 2013). VS species are not blind to UV light but lack the ability to distinguish between short wavelengths like UVS species (Emmerton & Delhis, 1980; Martin, 2022). The relatively low transmission of UV light in VS species is likely from the presence of UV‐blocking pigmentation in the eye lens (Lind et al., 2014).

Plants can increase signaling success to birds by enhancing chromatic (wavelength‐related) and/or achromatic (intensity‐related) contrasts between fruits/flowers and their backgrounds (Schaefer et al., 2006). Phenolics help plants visually guide pollinators to flowers (Iwashina, 2003) and later attract animal consumers to fruits (Samanta et al., 2011). Anthocyanins are colored flavonoids responsible for most of the red, pink, purple, and blue plant coloration (Taiz & Zeiger, 2006). Almost all polyphenols can act as co‐pigments, forming complexes with and changing the intensity or coloration of anthocyanins (Lattanzio et al., 2009). Distributions of flavonoids among flower tissues can create conspicuous patterns that help guide pollinators to nectar and pollen because they absorb UV light (Lunau, 1992). As fruits ripen, antioxidant activity and total phenolic content decrease (Castrejón et al., 2008). This decline in phenolics could explain why many fruits tend to reflect UV more after ripening (Cronin & Bok, 2016). There are exceptions to this trend. For example, as yew berries (*Taxus baccata*) ripen and change from green to red, UV reflectance declines (Cuthill et al., 2000).

Fruit color can act as an honest signal, allowing bird dispersers to increase their intake of vital nutrients and energy (Schaefer et al., 2014). Color change during ripening is considered an adaptation to attract seed dispersers (Valenta et al., 2017). Despite most bird‐dispersed fruits being red or black in color, this coloration alone does not seem to produce a significant preference (Willson et al., 1990). The UV color of a fruit's waxy cuticles makes the fruit more conspicuous to a bird on the lookout for its next meal (Burkhardt, 1982) because common natural backgrounds like leaves, bark, and soil do not reflect UV (Chittka et al., 1994). This increased UV reflectance makes the fruit brighter overall and does not produce a specific UV hue (Willson & Whelan, 1989). On the contrary, if the fruit absorbs UV more strongly relative to its background, conspicuousness can also increase (Bennett & Cuthill, 1994). The UV contrast of fruit against backgrounds is often enhanced by secondary structures like bracts and stems (Schmidt et al., 2004).

The use of UVS vision for foraging is most well‐known among frugivores. Fruits that reflect UV are strongly associated with the presence of UVS bird species (Rajchard, 2009). In Panama, a study by Altshuler (2001) showed that when a UV‐absorbing filter was used, foraging birds removed less fruits from shrubs. Birds in temperate regions also show fruit preference based on UV cues. Lab experiments show redwings (*Turdus iliacus*) and black grouse (*Lyrurus tetrix*) prefer UV‐reflecting blueberries (*Vaccinium myrtillus*) over non‐reflecting blueberries under natural conditions but have no preference when UV illumination is absent (Siitari et al., 1999, 2002). Fish crows (*Corvus ossifragus*) are able to detect UV‐reflecting blueberries from farther distances because of the fruits' increased intensity and color contrast with foliage (Schaefer et al., 2006). Eurasian blackcaps (*Sylvia atricapilla*) discriminate between the nutritional value (anthocyanin content) of fruits by selecting fruits based on their UV reflectance (Schaefer et al., 2008). In this sense, UV reflectance is an honest signal of nutrition since birds are actively selecting for higher anthocyanin content which is positively correlated with caloric value.

## 8 EVOLUTION OF FRUIT SIGNALS AND UVS VISION

Did preferences for UV‐contrast in fruits among UVS species come about through coevolution between birds and angiosperms? Or are these preferences just selection on the birds' behalf through the course of avian evolution?

The exact origin of angiosperms remains a mystery to this day (Li et al., 2019). Paleobotanists generally agree that the diversification of extant angiosperm lineages evolved during the Late Jurassic and Early Cretaceous Periods approximately 150 million years ago (Mya) (De Bodt et al., 2005). More than 80% of extant angiosperm orders likely originated during an early burst of diversification in the Early Cretaceous known as “Darwin's abominable mystery” (Zuntini et al., 2024). During the first 70–80 million years of their evolution, angiosperms had small fruits and seeds (Crane et al., 1995). The most important dispersal agents for these early fruits were likely rodent‐like mammals, the multituberculates, that are now extinct (Wilson et al., 2012). Significant diversification of angiosperm fruits began 80 Mya (Lupia et al., 1999) with rodents and primates taking over as the primary seed dispersers (Boyer et al., 2012; Collinson & Hooker, 2000). Diversification of fruit sizes and types peaked during the Eocene (55–50 Mya) (Eriksson et al., 2000). Flight became particularly advantageous after a climate shift around the Eocene–Oligocene boundary (34 Mya) which created patchy, semi‐open woodland habitats with food sources farther apart (Graham, 1999). Bats and birds, specifically passerines, became the dominant frugivores during the Oligocene (34–23 Mya) and Miocene (23–5 Mya), exploiting an already diverse variety of fleshy fruits (Mayr, 2005; Teeling et al., 2005).

Tetrachromacy and UVS pigment are the ancestral condition of tetrapods, with separate losses of UV sensitivity in mammals, amphibians, and birds (Hunt et al., 2001). The ancestor of passerines is hypothesized to have had UVS pigment (Ödeen et al., 2011) with initial passerine divergence occurring 71–60 Mya (Ericson et al., 2002). Passerine diversification rates significantly increased during the Late Miocene (Oliveros et al., 2019). Today, passerines are the most common and widespread frugivores in the animal kingdom. Because tetrachromacy preceded the radiation of many angiosperms, it is likely that fruit color is an evolved attractant to birds (Valenta et al., 2017). Color of bird‐dispersed fruits has experienced a stronger selection for conspicuousness than that of mammal‐dispersed fruits (Duan et al., 2014; Lomascolo & Schaefer, 2010). Despite this, the Oligocene–Miocene “passerine take‐over” was most likely not because of coevolution between birds and fruits. Passerines were able to exploit an existing niche under new conditions, but the fruits evolved through interactions with extinct mammals (Eriksson, 2016). It is clear the success of passerines was not only related to frugivory (Jønsson et al., 2011).

There is a general lack of evidence for the evolution of fruit color being driven by avian frugivore selection (Wheelwright & Janson, 1985). Flowers show strong coevolution with pollinators. Because the plant requires pollen to be taken to a conspecific, selection has been towards food‐rewarding flowers that attract a narrow range of pollinators (Johnson & Anderson, 2010). Coevolution between frugivores and plants, however, is less likely. Fruits benefit from interacting with a large variety of frugivores (Schaefer et al., 2004). Similarly, frugivores consume many different fruits and their dependence on a particular species is insignificant (Herrera, 2002; Jordano, 2000). Therefore, fruit color is expected to converge towards similar coloration that can be easily detected by a wide range of animals (Valenta et al., 2015). Modeling fruit reflectance according to avian vision shows selection for increased detectability is not the primary driver of fruit color (Schaefer et al., 2007). Other selective pressures that may influence the evolution of UV‐reflecting pigmentation include pathogen defense and reduction of desiccation (Schaefer & Schaefer, 2007). Phenolics may be selected for by frugivores and by fruits alike for their antioxidant properties (Schaefer, 2011).

## 9 CONCLUSIONS

The origin of phenolics in early land plants is closely tied to their ability to absorb UV light and antioxidant properties. As plants evolved, phenolics diversified, becoming important in defense against pathogens and herbivores. Phenolics are also involved in plant reproduction, regulating seed dormancy, attracting pollinators to flowers, and enticing seed dispersers with colorful fruit. Endozoochorous plant species benefit from attracting frugivores by movement and dispersal of seeds as well as increased germinability after passing through the animal digestive tract. Birds are perhaps the most common frugivores and use UVS vision to detect ripe fruit with their contrasting backgrounds. Changes in UV reflection as fruits ripen are linked to phenolic content in the fruits' waxy outer layers. However, it is likely that the evolution of UV‐contrast in fruits is not due to coevolution with birds. Passerines exploited an existing niche of fruit‐producing plants that evolved through interactions with extinct mammals. Coevolution between seed dispersers and fruits is less likely than between pollinators and flowers because plants benefit from interacting with an increasing variety of frugivores. Despite this, modern passerines often still appear to use UV cues that evolved long ago to select fruits based on phenolic content.

This review has highlighted new research opportunities and identified gaps in current scientific understanding. Most studies on fruit selection and the effects of endozoochory on seeds have been conducted in laboratory settings. Field studies could expand the ecological context of these interactions by considering factors such as climate, habitat, competitors, predators, and natural food sources. There is a significant lack of comprehensive data on the phenolic content in seeds dispersed by birds, particularly before and after gut passage. Examining the chemical content of seeds before and after endozoochory, alongside measuring germination rates, could provide more insight into which phenolics are most influential on germination and/or most sensitive to digestion. Lastly, there is limited understanding of the conservation implications of phenolic‐mediated seed dispersal. The role of phenolics in attracting bird dispersers and their effect on germination could potentially influence the success of invasive and/or introduced fruit‐bearing species, such as rosa multiflora (Liaudanskas et al., 2021).

## AUTHOR CONTRIBUTIONS

**Samuel A. Krebs:** Conceptualization (equal); investigation (lead); project administration (equal); writing – original draft (lead). **Michael L. Schummer:** Conceptualization (equal); project administration (equal); writing – original draft (supporting).

## CONFLICT OF INTEREST STATEMENT

The authors declare that they have no known competing financial interests or personal relationships that could have appeared to influence the work reported in this paper.

## Data Availability

This is a review article; all research papers used by the authors are included in the literature cited. No data sets were used by the authors.

## References

[ece370255-bib-0001] Abenavoli, M. R. , Cacco, G. , Sorgona, A. , Marabottini, R. , Paolacci, A. R. , Ciaffi, M. , & Badiani, M. (2006). The inhibitory effects of coumarin on the germination of durum wheat (*Triticum turgidum* ssp. durum, cv. Simeto) seeds. Journal of Chemical Ecology, 32, 489–506. 16598652 10.1007/s10886-005-9011-x

[ece370255-bib-0002] Al Mamari, H. H. (2021). Phenolic compounds: Classification, chemistry, and updated techniques of analysis and synthesis. Phenolic Compounds: Chemistry, Synthesis, Diversity, Non‐Conventional Industrial, Pharmaceutical and Therapeutic Applications, 10, 73–94.

[ece370255-bib-0003] Altshuler, D. L. (2001). Ultraviolet reflectance in fruits, ambient light composition and fruit removal in a tropical forest. Evolutionary Ecology Research, 3, 767–778.

[ece370255-bib-0004] Apel, K. , & Hirt, H. (2004). Reactive oxygen species: Metabolism, oxidative stress, and signal transduction. Annual Review of Plant Biology, 55, 373–399. 10.1146/annurev.arplant.55.031903.141701 15377225

[ece370255-bib-0005] Barbehenn, R. V. , & Constabel, C. P. (2011). Tannins in plant–herbivore interactions. Phytochemistry, 72, 1551–1565. 21354580 10.1016/j.phytochem.2011.01.040

[ece370255-bib-0006] Barnea, A. , Yom‐Tov, Y. , & Friedman, J. (1991). Does ingestion by birds affect seed germination? Functional Ecology, 5, 394–402.

[ece370255-bib-0007] Baskin, J. M. , & Baskin, C. C. (2004). A classification system for seed dormancy. Seed Science Research, 14, 1–16.

[ece370255-bib-0008] Baskin, J. M. , & Baskin, C. C. (2021). The great diversity in kinds of seed dormancy: A revision of the Nikolaeva–Baskin classification system for primary seed dormancy. Seed Science Research, 31, 249–277.

[ece370255-bib-0009] Becerra, J. X. , Noge, K. , & Venable, D. L. (2009). Macroevolutionary chemical escalation in an ancient plant–herbivore arms race. Proceedings of the National Academy of Sciences, 106(43), 18062–18066. 10.1073/pnas.0904456106 PMC2775328 19706441

[ece370255-bib-0010] Bennett, A. T. , & Cuthill, I. C. (1994). Ultraviolet vision in birds: What is its function? Vision Research, 34, 1471–1478. 8023459 10.1016/0042-6989(94)90149-x

[ece370255-bib-0011] Bennett, R. N. , & Wallsgrove, R. M. (1994). Secondary metabolites in plant defence mechanisms. New Phytologist, 127, 617–633. 33874382 10.1111/j.1469-8137.1994.tb02968.x

[ece370255-bib-0012] Bewley, J. D. (1997). Seed germination and dormancy. The Plant Cell, 9, 1055–1066. 12237375 10.1105/tpc.9.7.1055 PMC156979

[ece370255-bib-0013] Botha, F. C. , Potgieter, G. P. , & Botha, A. M. (1992). Respiratory metabolism and gene expression during seed germination. Plant Growth Regulation, 11, 211–224.

[ece370255-bib-0014] Boyer, D. M. , Costeur, L. , & Lipman, Y. (2012). Earliest record of *Platychoerops* (primates, *Plesiadapidae*), a new species from Mouras quarry, Mont de Berru, France. American Journal of Physical Anthropology, 149, 329–346. 22926965 10.1002/ajpa.22119

[ece370255-bib-0015] Brooker, N. , Windorski, J. , & Bluml, E. (2008). Halogenated coumarin derivatives as novel seed protectants. Communications in Agricultural and Applied Biological Sciences, 73(2), 81–89. 19226745

[ece370255-bib-0016] Bryant, J. P. (1981). Phytochemical deterrence of snowshoe hare browsing by adventitious shoots of four Alaskan trees. Science, 213, 889–890. 17775273 10.1126/science.213.4510.889

[ece370255-bib-0017] Burkhardt, D. (1982). Birds, berries, and UV: A note on some consequences of UV vision in birds. Naturwissenschaften, 69, 153–157. 7088195 10.1007/BF00364887

[ece370255-bib-0018] Burns, K. J. , & Shultz, A. J. (2012). Widespread cryptic dichromatism and ultraviolet reflectance in the largest radiation of Neotropical songbirds: Implications of accounting for avian vision in the study of plumage evolution. The Auk, 129, 211–221.

[ece370255-bib-0019] Butler, L. G. (1989). Effects of condensed tannin on animal nutrition. In Chemistry and significance of condensed tannins (pp. 391–402). Springer.

[ece370255-bib-0020] Campos, C. M. , & Ojeda, R. A. (1997). Dispersal and germination of *Prosopis flexuosa* (*Fabaceae*) seeds by desert mammals in Argentina. Journal of Arid Environments, 35, 707–714.

[ece370255-bib-0021] Campos, C. M. , Ramos, L. , Manrique, N. , Cona, M. I. , Sartor, C. , de los Ríos, C. , & Cappa, F. M. (2020). Filling gaps in the seed dispersal effectiveness model for *Prosopis flexuosa*: Quality of seed treatment in the digestive tract of native animals. Seed Science Research, 30, 215–223.

[ece370255-bib-0022] Castrejón, A. D. R. , Eichholz, I. , Rohn, S. , Kroh, L. W. , & Huyskens‐Keil, S. (2008). Phenolic profile and antioxidant activity of highbush blueberry (*Vaccinium corymbosum* L.) during fruit maturation and ripening. Food Chemistry, 109, 564–572.

[ece370255-bib-0023] Chaki, M. , Begara‐Morales, J. C. , & Barroso, J. B. (2020). Oxidative stress in plants. Antioxidants, 9, 481. 32503179 10.3390/antiox9060481 PMC7346199

[ece370255-bib-0024] Charalambidou, I. , Santamaría, L. , Jansen, C. , & Nolet, B. A. (2005). Digestive plasticity in mallard ducks modulates dispersal probabilities of aquatic plants and crustaceans. Functional Ecology, 19, 513–519.

[ece370255-bib-0025] Charalambidou, I. , Santamaria, L. , & Langevoord, O. (2003). Effect of ingestion by five avian dispersers on the retention time, retrieval, and germination of *Ruppia maritima* seeds. Functional Ecology, 17, 747–753.

[ece370255-bib-0026] Cheynier, V. , Comte, G. , Davies, K. M. , Lattanzio, V. , & Martens, S. (2013). Plant phenolics: Recent advances on their biosynthesis, genetics, and ecophysiology. Plant Physiology and Biochemistry, 72, 1–20. 23774057 10.1016/j.plaphy.2013.05.009

[ece370255-bib-0027] Chittka, L. , Shmida, A. V. I. , Troje, N. , & Menzel, R. (1994). Ultraviolet as a component of flower reflections, and the colour perception of *Hymenoptera* . Vision Research, 34, 1489–1508. 8023461 10.1016/0042-6989(94)90151-1

[ece370255-bib-0028] Collinson, M. E. , & Hooker, J. J. (2000). Gnaw marks on Eocene seeds: Evidence for early rodent behaviour. Palaeogeography, Palaeoclimatology, Palaeoecology, 157, 127–149.

[ece370255-bib-0029] Cooper‐Driver, G. A. , & Bhattacharya, M. (1998). Role of phenolics in plant evolution. Phytochemistry, 49, 1165–1174.

[ece370255-bib-0030] Corso, M. , Perreau, F. , Mouille, G. , & Lepiniec, L. (2020). Specialized phenolic compounds in seeds: Structures, functions, and regulations. Plant Science, 296, 110471. 32540001 10.1016/j.plantsci.2020.110471

[ece370255-bib-0031] Cosyns, E. , Delporte, A. , Lens, L. , & Hoffmann, M. (2005). Germination success of temperate grassland species after passage through ungulate and rabbit guts. Journal of Ecology, 93, 353–361.

[ece370255-bib-0032] Counet, C. , Callemien, D. , & Collin, S. (2006). Chocolate and cocoa: New sources of trans‐resveratrol and trans‐piceid. Food Chemistry, 98(4), 649–657.

[ece370255-bib-0033] Crane, P. R. , Friis, E. M. , & Pedersen, K. R. (1995). The origin and early diversification of angiosperms. Nature, 374, 27–33.

[ece370255-bib-0034] Cronin, T. W. , & Bok, M. J. (2016). Photoreception and vision in the ultraviolet. Journal of Experimental Biology, 219, 2790–2801. 27655820 10.1242/jeb.128769

[ece370255-bib-0035] Cuadra, P. , & Harborne, J. B. (1996). Changes in epicuticular flavonoids and photosynthetic pigments as a plant response to UV‐B radiation. Zeitschrift für Naturforschung. Section C, 51, 671–680.

[ece370255-bib-0036] Cuthill, I. (2006). Color perception. In G. E. Hill & K. J. McGraw (Eds.), Bird coloration. Vol. 1. Mechanisms and measurements (pp. 3–40). Harvard University Press.

[ece370255-bib-0037] Cuthill, I. C. , Partridge, J. C. , Bennett, A. T. , Church, S. C. , Hart, N. S. , & Hunt, S. (2000). Ultraviolet vision in birds. In Advances in the study of behavior, 29 (pp. 159–214). Academic Press.

[ece370255-bib-0038] De Bodt, S. , Maere, S. , & Van de Peer, Y. (2005). Genome duplication and the origin of angiosperms. Trends in Ecology & Evolution, 20, 591–597. 16701441 10.1016/j.tree.2005.07.008

[ece370255-bib-0039] Debeaujon, I. , Leon‐Kloosterziel, K. M. , & Koornneef, M. (2000). Influence of the testa on seed dormancy, germination, and longevity in Arabidopsis. Plant Physiology, 122(2), 403–414. 10677433 10.1104/pp.122.2.403 PMC58877

[ece370255-bib-0040] Delaux, P. M. , Nanda, A. K. , Mathé, C. , Sejalon‐Delmas, N. , & Dunand, C. (2012). Molecular and biochemical aspects of plant terrestrialization. Perspectives in Plant Ecology, Evolution and Systematics, 14, 49–59.

[ece370255-bib-0041] Duan, Q. , Goodale, E. , & Quan, R. C. (2014). Bird fruit preferences match the frequency of fruit colours in tropical Asia. Scientific Reports, 4, 5627. 25033283 10.1038/srep05627 PMC4102077

[ece370255-bib-0042] Emmerton, J. , & Delhis, J. D. (1980). Wavelength discrimination in the visible and ultraviolet spectrum by pigeons. Journal of Comparative Physiology, 14, 47–52.

[ece370255-bib-0043] Ericson, P. G. , Christidis, L. , Cooper, A. , Irestedt, M. , Jackson, J. , Johansson, U. S. , & Norman, J. A. (2002). A Gondwanan origin of passerine birds supported by DNA sequences of the endemic New Zealand wrens. Proceedings of the Royal Society of London. Series B: Biological Sciences, 269, 235–241. 10.1098/rspb.2001.1877 PMC1690883 11839192

[ece370255-bib-0044] Eriksson, O. (2016). Evolution of angiosperm seed disperser mutualisms: The timing of origins and their consequences for coevolutionary interactions between angiosperms and frugivores. Biological Reviews, 91, 168–186. 25530412 10.1111/brv.12164

[ece370255-bib-0045] Eriksson, O. , Friis, E. M. , & Löfgren, P. (2000). Seed size, fruit size, and dispersal systems in angiosperms from the Early Cretaceous to the late tertiary. The American Naturalist, 156, 47–58. 10.1086/303367 10824020

[ece370255-bib-0046] Figueroa, J. G. , Borrás‐Linares, I. , Lozano‐Sánchez, J. , & Segura‐Carretero, A. (2018). Comprehensive characterization of phenolic and other polar compounds in the seed and seed coat of avocado by HPLC‐DAD‐ESI‐QTOF‐MS. Food Research International, 105, 752–763. 29433270 10.1016/j.foodres.2017.11.082

[ece370255-bib-0047] Gharachorloo, M. , Ghiassi Tarzi, B. , & Baharinia, M. (2013). The effect of germination on phenolic compounds and antioxidant activity of pulses. Journal of the American Oil Chemists' Society, 90, 407–411.

[ece370255-bib-0048] Goldsmith, T. H. (1990). Optimization, constraint, and history in the evolution of eyes. The Quarterly Review of Biology, 65, 281–322. 2146698 10.1086/416840

[ece370255-bib-0049] Goldstein, J. L. , & Swain, T. (1963). Changes in tannins in ripening fruits. Phytochemistry, 2, 371–383.

[ece370255-bib-0050] Grace, S. C. (2005). Phenolics as antioxidants. Antioxidants and Reactive Oxygen Species in Plants, 141–168.

[ece370255-bib-0051] Graham, A. (1999). Late cretaceous and Cenozoic history of north American vegetation: North of Mexico. Oxford University Press.

[ece370255-bib-0052] Graham, L. E. , Kodner, R. B. , Fisher, M. M. , Graham, J. M. , Wilcox, L. W. , Hackney, J. M. , Obst, J. , Bilkey, P. C. , Hanson, D. T. , & Cook, M. E. (2004). Early land plant adaptations to terrestrial stress: A focus on phenolics. In The evolution of plant physiology (p. 155–169). Academic Press.

[ece370255-bib-0053] Guerrero‐Castillo, P. , Reyes, S. , Robles, J. , Simirgiotis, M. J. , Sepulveda, B. , Fernandez‐Burgos, R. , & Areche, C. (2019). Biological activity and chemical characterization of *Pouteria lucuma* seeds: A possible use of an agricultural waste. Waste Management, 88, 319–327. 31079645 10.1016/j.wasman.2019.03.055

[ece370255-bib-0054] Hammerschmidt, R. (1999). Phytoalexins: What have we learned after 60 years? Annual Review of Phytopathology, 37, 285–306. 10.1146/annurev.phyto.37.1.285 11701825

[ece370255-bib-0055] Harborne, J. B. (1990). Constraints on the evolution of biochemical pathways. Biological Journal of the Linnean Society, 39, 135–151.

[ece370255-bib-0056] Hart, N. S. (2001). The visual ecology of avian photoreceptors. Progress in Retinal and Eye Research, 20, 675–703. 11470455 10.1016/s1350-9462(01)00009-x

[ece370255-bib-0057] Hasan, M. M. , & Bae, H. (2017). An overview of stress‐induced resveratrol synthesis in grapes: Perspectives for resveratrol‐enriched grape products. Molecules, 22(2), 294. 28216605 10.3390/molecules22020294 PMC6155908

[ece370255-bib-0058] Herms, D. A. , & Mattson, W. J. (1992). The dilemma of plants: To grow or defend. The Quarterly Review of Biology, 67, 283–335.

[ece370255-bib-0059] Herrera, C. M. (1989). Seed dispersal by animals: A role in angiosperm diversification? The American Naturalist, 133, 309–322.

[ece370255-bib-0060] Herrera, C. M. (2002). Seed dispersal by vertebrates. In C. M. Herrera & O. Pellmyr (Eds.), Plant–animal interactions: An evolutionary approach (pp. 185–208). Blackwell Science.

[ece370255-bib-0061] Herrera, C. M. , & Pellmyr, O. (Eds.). (2009). Plant animal interactions: An evolutionary approach. John Wiley & Sons.

[ece370255-bib-0062] Hoshino, T. , & Duncan, R. R. (1982). Sorghum tannin content during maturity under different environmental conditions. Japanese Journal of Crop Science, 51, 178–184.

[ece370255-bib-0063] Hunt, D. M. , Wilkie, S. E. , Bowmaker, J. K. , & Poopalasundaram, S. (2001). Vision in the ultraviolet. Cellular and Molecular Life Sciences, 58, 1583–1598. 11706986 10.1007/PL00000798 PMC11337280

[ece370255-bib-0064] Husband, S. , & Shimizu, T. (2001). Evolution of the avian vision.

[ece370255-bib-0065] Igamberdiev, A. U. , & Lea, P. J. (2006). Land plants equilibrate O_2_ and CO_2_ concentrations in the atmosphere. Photosynthesis Research, 87, 177–194. 16432665 10.1007/s11120-005-8388-2

[ece370255-bib-0066] Iiyama, K. , Lam, T. B. T. , & Stone, B. A. (1994). Covalent cross‐links in the cell wall. Plant Physiology, 104, 315–320. 12232082 10.1104/pp.104.2.315 PMC159201

[ece370255-bib-0067] Iluz, D. (2011). Zoochory: The dispersal of plants by animals. In Z. Dubinsky & J. Seckbach (Eds.), All flesh ts grass: Plant–animal interrelationships (pp. 199–214). Springer.

[ece370255-bib-0068] Inácio, M. C. , Moraes, R. M. , Mendonça, P. C. , Morel, L. J. , França, S. C. , Bertoni, B. W. , & Pereira, A. M. (2013). Phenolic compounds influence seed dormancy of *Palicourea rigida HBK* (*Rubiaceae*), a medicinal plant of the Brazilian Savannah.

[ece370255-bib-0069] Iwashina, T. (2003). Flavonoid function and activity to plants and other organisms. Biological Sciences in Space, 17, 24–44. 12897458 10.2187/bss.17.24

[ece370255-bib-0070] Jakubas, W. J. , & Gullion, G. W. (1991). Use of quaking aspen flower buds by ruffed grouse: Its relationship to grouse densities and bud chemical composition. The Condor, 93, 473–485.

[ece370255-bib-0071] Johnson, S. D. , & Anderson, B. (2010). Coevolution between food‐rewarding flowers and their pollinators. Evolution: Education and Outreach, 3, 32–39.

[ece370255-bib-0072] Jones, M. P. , Pierce, K. E., Jr. , & Ward, D. (2007). Avian vision: A review of form and function with special consideration to birds of prey. Journal of Exotic Pet Medicine, 16, 69–87.

[ece370255-bib-0073] Jønsson, K. A. , Fabre, P. H. , Ricklefs, R. E. , & Fjeldså, J. (2011). Major global radiation of corvoid birds originated in the proto‐Papuan archipelago. Proceedings of the National Academy of Sciences, 108, 2328–2333. 10.1073/pnas.1018956108 PMC3038755 21262814

[ece370255-bib-0074] Jordano, P. (2000). Fruits and frugivory. Seeds: The Ecology of Regeneration in Plant Communities, 2, 125–166.

[ece370255-bib-0075] Jung, H. G. , & Allen, M. S. (1995). Characteristics of plant cell walls affecting intake and digestibility of forages by ruminants. Journal of Animal Science, 73, 2774–2790. 8582870 10.2527/1995.7392774x

[ece370255-bib-0076] Karabourniotis, G. , Liakopoulos, G. , Nikolopoulos, D. , Bresta, P. , Stavroulaki, V. , & Sumbele, S. (2014). “Carbon gain vs. water saving, growth vs. defence”: Two dilemmas with soluble phenolics as a joker. Plant Science, 227, 21–27. 25219302 10.1016/j.plantsci.2014.06.014

[ece370255-bib-0077] Keen, N. T. (1981). Evaluation of the role of phytoalexins. In R. C. Staples & G. H. Toenniessen (Eds.), Plant disease control (pp. 155–177). Wiley.

[ece370255-bib-0078] Kimura, E. , & Islam, M. A. (2012). Seed scarification methods and their use in forage legumes. Research Journal of Seed Science, 5, 38–50.

[ece370255-bib-0079] Koornneef, M. , Bentsink, L. , & Hilhorst, H. (2002). Seed dormancy and germination. Current Opinion in Plant Biology, 5, 33–36. 11788305 10.1016/s1369-5266(01)00219-9

[ece370255-bib-0080] Koricheva, J. (2002). Meta‐analysis of sources of variation in fitness costs of plant antiherbivore defenses. Ecology, 83(1), 176–190.

[ece370255-bib-0081] Krefting, L. W. , & Roe, E. I. (1949). The role of some birds and mammals in seed germination. Ecological Monographs, 19, 269–286.

[ece370255-bib-0082] Lattanzio, V. , Kroon, P. A. , Quideau, S. , & Treutter, D. (2009). Plant phenolics—Secondary metabolites with diverse functions. Recent Advances in Polyphenol Research, 1, 1–35.

[ece370255-bib-0083] Lee, J. , Hwang, Y. S. , Kim, S. T. , Yoon, W. B. , Han, W. Y. , Kang, I. K. , & Choung, M. G. (2017). Seed coat color and seed weight contribute differential responses of targeted metabolites in soybean seeds. Food Chemistry, 214, 248–258. 27507473 10.1016/j.foodchem.2016.07.066

[ece370255-bib-0084] Lepiniec, L. , Debeaujon, I. , Routaboul, J. M. , Baudry, A. , Pourcel, L. , Nesi, N. , & Caboche, M. (2006). Genetics and biochemistry of seed flavonoids. Annual Review of Plant Biology, 57, 405–430. 10.1146/annurev.arplant.57.032905.105252 16669768

[ece370255-bib-0085] Li, H. T. , Yi, T. S. , Gao, L. M. , Ma, P. F. , Zhang, T. , Yang, J. B. , Gitzendanner, M. A. , Fritsch, P. W. , Cai, J. , Luo, Y. , & Wang, H. (2019). Origin of angiosperms and the puzzle of the Jurassic gap. Nature Plants, 5, 461–470. 31061536 10.1038/s41477-019-0421-0

[ece370255-bib-0086] Li, Z. H. , Wang, Q. , Ruan, X. , Pan, C. D. , & Jiang, D. A. (2010). Phenolics and plant allelopathy. Molecules, 15, 8933–8952. 21139532 10.3390/molecules15128933 PMC6259130

[ece370255-bib-0087] Liaudanskas, M. , Noreikienė, I. , Zymonė, K. , Juodytė, R. , Žvikas, V. , & Janulis, V. (2021). Composition and antioxidant activity of phenolic compounds in fruit of the genus *Rosa* L. Antioxidants, 10(4), 545. 33915934 10.3390/antiox10040545 PMC8065802

[ece370255-bib-0088] Lind, O. , Mitkus, M. , Olsson, P. , & Kelber, A. (2014). Ultraviolet vision in birds: The importance of transparent eye media. Proceedings of the Royal Society B: Biological Sciences, 281(1774), 20132209. 10.1098/rspb.2013.2209 PMC3843830 24258716

[ece370255-bib-0089] Lindroth, R. L. , & Hwang, S. Y. (1996). Diversity, redundancy, and multiplicity in chemical defense systems of aspen. In Phytochemical diversity and redundancy in ecological interactions (pp. 25–56). Springer.

[ece370255-bib-0090] Lomascolo, S. B. , & Schaefer, H. M. (2010). Signal convergence in fruits: A result of selection by frugivores? Journal of Evolutionary Biology, 23, 614–624. 20487134 10.1111/j.1420-9101.2010.01931.x

[ece370255-bib-0091] Lunau, K. (1992). A new interpretation of flower guide colouration: Absorption of ultraviolet light enhances colour saturation. Plant Systematics and Evolution, 183, 51–65.

[ece370255-bib-0092] Lupia, R. , Lidgard, S. , & Crane, P. R. (1999). Comparing palynological abundance and diversity: Implications for biotic replacement during the cretaceous angiosperm radiation. Paleobiology, 25, 305–340.

[ece370255-bib-0093] MacGregor, D. R. , Kendall, S. L. , Florance, H. , Fedi, F. , Moore, K. , Paszkiewicz, K. , Smirnoff, N. , & Penfield, S. (2015). Seed production temperature regulation of primary dormancy occurs through control of seed coat phenylpropanoid metabolism. New Phytologist, 205(2), 642–652. 25412428 10.1111/nph.13090

[ece370255-bib-0094] Markham, K. R. (1990). Bryophyte flavonoids, their structures, distribution, and evolutionary significance. In H. D. Zinsmeister & R. Mues (Eds.), Bryophytes: Their chemistry and chemical taxonomy (pp. 143–159). Oxford University Press.

[ece370255-bib-0095] Markham, K. R. , Ryan, K. G. , Bloor, S. J. , & Mitchell, K. A. (1998). An increase in the luteolin: Apigenin ratio in *Marchantia polymorpha* on UV‐B enhancement. Phytochemistry, 48, 791–794.

[ece370255-bib-0096] Martin, G. R. (2007). Visual fields and their functions in birds. Journal of Ornithology, 148, 547–562.

[ece370255-bib-0097] Martin, G. R. (2022). Avian vision. Current Biology, 32, 1079–1085. 10.1016/j.cub.2022.06.065 36283369

[ece370255-bib-0098] Martin Schaefer, H. , Schaefer, V. , & Vorobyev, M. (2007). Are fruit colors adapted to consumer vision and birds equally efficient in detecting colorful signals? The American Naturalist, 169, 159–169. 10.1086/510097 19426090

[ece370255-bib-0099] Matos, M. J. , Santana, L. , Uriarte, E. , Abreu, O. A. , Molina, E. , & Yordi, E. G. (2015). Coumarins—An important class of phytochemicals. Phytochemicals‐Isolation, Characterisation and Role in Human Health, 25, 533–538.

[ece370255-bib-0100] Mayr, G. (2005). The Paleogene fossil record of birds in Europe. Biological Reviews, 80, 515–542. 16221327 10.1017/S1464793105006779

[ece370255-bib-0101] McMillian, W. W. , Wiseman, B. R. , Burns, R. E. , Harris, H. B. , & Greene, G. L. (1972). Bird resistance in diverse germplasm of *sorghum 1* . Agronomy Journal, 64, 821–822.

[ece370255-bib-0102] Moreland, D. E. , & Novitzky, W. P. (1987). Effects of phenolic acids, coumarins, and flavonoids on isolated chloroplasts and mitochondria.

[ece370255-bib-0103] Morpeth, D. R. , & Hall, A. M. (2000). Microbial enhancement of seed germination in *Rosa corymbifera* ‘*Laxa*’. Seed Science Research, 10, 489–494.

[ece370255-bib-0104] Mousavi, S. R. , Rezaei, M. , & Mousavi, A. (2011). A general overview on seed dormancy and methods of breaking it. Advances in Environmental Biology, 5, 3333–3337.

[ece370255-bib-0105] Muscolo, A. , Panuccio, M. R. , & Sidari, M. (2001). The effect of phenols on respiratory enzymes in seed germination. Plant Growth Regulation, 35, 31–35.

[ece370255-bib-0106] Mustafa, Y. F. , Najem, M. A. , & Tawffiq, Z. S. (2018). Coumarins from Creston apple seeds: Isolation, chemical modification, and cytotoxicity study. Journal of Applied Pharmaceutical Science, 8(8), 49–56.

[ece370255-bib-0107] Nogales, M. , Nieves, C. , Illera, J. C. , Padilla, D. P. , & Traveset, A. (2005). Effect of native and alien vertebrate frugivores on seed viability and germination patterns of *Rubia fruticosa* (*Rubiaceae*) in the eastern Canary Islands. Functional Ecology, 19, 429–436.

[ece370255-bib-0108] Ödeen, A. , & Håstad, O. (2013). The phylogenetic distribution of ultraviolet sensitivity in birds. BMC Evolutionary Biology, 13, 1–10. 23394614 10.1186/1471-2148-13-36 PMC3637589

[ece370255-bib-0109] Ödeen, A. , Håstad, O. , & Alström, P. (2011). Evolution of ultraviolet vision in the largest avian radiation‐the passerines. BMC Evolutionary Biology, 11, 1–8. 22024316 10.1186/1471-2148-11-313 PMC3225180

[ece370255-bib-0110] Oliveros, C. H. , Field, D. J. , Ksepka, D. T. , Barker, F. K. , Aleixo, A. , Andersen, M. J. , Alström, P. , Benz, B. W. , Braun, E. L. , Braun, M. J. , & Bravo, G. A. (2019). Earth history and the passerine superradiation. Proceedings of the National Academy of Sciences, 116, 7916–7925. 10.1073/pnas.1813206116 PMC6475423 30936315

[ece370255-bib-0111] Olsson, P. , Lind, O. , Mitkus, M. , Delhey, K. , & Kelber, A. (2021). Lens and cornea limit UV vision of birds–a phylogenetic perspective. Journal of Experimental Biology, 224(20), jeb243129. 34581400 10.1242/jeb.243129 PMC8601714

[ece370255-bib-0112] Pratyusha, S. (2022). Phenolic compounds in the plant development and defense: An overview. In M. Hasanuzzaman & K. Nahar (Eds.), Plant stress physiology‐perspectives in agriculture (pp. 125–140). IntechOpen.

[ece370255-bib-0113] Rajchard, J. (2009). Ultraviolet (UV) light perception by birds: A review. Veterinární Medicína, 54, 351–359.

[ece370255-bib-0114] Reichardt, P. B. , Bryant, J. P. , Mattes, B. R. , Clausen, T. P. , Chapin, F. S. , & Meyer, M. (1990). Winter chemical defense of Alaskan balsam poplar against snowshoe hares. Journal of Chemical Ecology, 16, 1941–1959. 24263997 10.1007/BF01020507

[ece370255-bib-0115] Rick, C. M. , & Bowman, R. I. (1961). Galapagos tomatoes and tortoises. Evolution, 15, 407–417.

[ece370255-bib-0116] Samanta, A. , Das, G. , & Das, S. K. (2011). Roles of flavonoids in plants. Carbon, 100, 12–35.

[ece370255-bib-0117] Santiago, L. J. M. , Louro, R. P. , & De Oliveira, D. E. (2000). Compartmentation of phenolic compounds and phenylalanine ammonia‐lyase in leaves of *Phyllanthus tenellus Roxb*. and their induction by copper sulphate. Annals of Botany, 86, 1023–1032.

[ece370255-bib-0118] Scalbert, A. (1992). Tannins in woods and their contribution to microbial decay prevention. In Plant polyphenols: Synthesis, properties, significance (pp. 935–952). Springer.

[ece370255-bib-0119] Schaefer, H. M. (2011). Why fruits go to the dark side. Acta Oecologica, 37, 604–610.

[ece370255-bib-0120] Schaefer, H. M. , Levey, D. J. , Schaefer, V. , & Avery, M. L. (2006). The role of chromatic and achromatic signals for fruit detection by birds. Behavioral Ecology, 17, 784–789.

[ece370255-bib-0121] Schaefer, H. M. , McGraw, K. , & Catoni, C. (2008). Birds use fruit colour as honest signal of dietary antioxidant rewards. Functional Ecology, 22, 303–310.

[ece370255-bib-0122] Schaefer, H. M. , & Schaefer, V. (2007). The evolution of visual fruit signals: Concepts and constraints. In Seed dispersal: Theory and its application in a changing world (pp. 59–77). CABI.

[ece370255-bib-0123] Schaefer, H. M. , Schaefer, V. , & Levey, D. J. (2004). How plant–animal interactions signal new insights in communication. Trends in Ecology & Evolution, 19, 577–584.

[ece370255-bib-0124] Schaefer, H. M. , Valido, A. , & Jordano, P. (2014). Birds see the true colours of fruits to live off the fat of the land. Proceedings of the Royal Society B: Biological Sciences, 281(1777), 20132516. 10.1098/rspb.2013.2516 PMC3896014 24403330

[ece370255-bib-0125] Schmidt, V. , Martin Schaefer, H. , & Winkler, H. (2004). Conspicuousness, not colour as foraging cue in plant–animal signalling. Oikos, 106, 551–557.

[ece370255-bib-0126] Schmutz, A. , Buchala, A. , Ryser, U. , & Jenny, T. (1993). The phenols in the wax and in the suberin polymer of green cotton fibres and their functions. International Symposium on Natural Phenols in Plant Resistance, 381, 269–275.

[ece370255-bib-0127] Schupp, E. W. (1993). Quantity, quality and the effectiveness of seed dispersal by animals. Vegetatio, 107, 15–29.

[ece370255-bib-0128] Shirley, B. W. (1998). Flavonoids in seeds and grains: Physiological function, agronomic importance and the genetics of biosynthesis. Seed Science Research, 8(4), 415–422.

[ece370255-bib-0129] Siitari, H. , Honkavaara, J. , & Viitala, J. (1999). Ultraviolet reflection of berries attracts foraging birds. A laboratory study with redwings (*Turdus iliacus*) and bilberries (*Vaccinium myrtillus*). Proceedings of the Royal Society of London. Series B: Biological Sciences, 266, 2125–2129.

[ece370255-bib-0130] Siitari, H. , Viitala, J. , & Hovi, M. (2002). Behavioural evidence for ultraviolet vision in a tetraonid species–foraging experiment with black grouse *Tetrao tetrix* . Journal of Avian Biology, 33, 199–202.

[ece370255-bib-0131] Singh, B. , Singh, J. P. , Kaur, A. , & Singh, N. (2017). Phenolic composition and antioxidant potential of grain legume seeds: A review. Food Research International, 101, 1–16. 28941672 10.1016/j.foodres.2017.09.026

[ece370255-bib-0132] Soltani, E. , Baskin, C. C. , Baskin, J. M. , Heshmati, S. , & Mirfazeli, M. S. (2018). A meta‐analysis of the effects of frugivory (endozoochory) on seed germination: Role of seed size and kind of dormancy. Plant Ecology, 219, 1283–1294.

[ece370255-bib-0133] Stafford, H. A. (2000). The evolution of phenolics in plants. Recent Advances in Phytochemistry, 34, 25–54.

[ece370255-bib-0134] Sumbele, S. , Fotelli, M. N. , Nikolopoulos, D. , Tooulakou, G. , Liakoura, V. , Liakopoulos, G. , Bresta, P. , Dotsika, E. , Adams, M. A. , & Karabourniotis, G. (2012). Photosynthetic capacity is negatively correlated with the concentration of leaf phenolic compounds across a range of different species. Oxford University Press. 10.1093/aobpla/pls025 PMC3465559 23050073

[ece370255-bib-0135] Swank, W. G. (1944). Germination of seeds after ingestion by ring‐necked pheasants. The Journal of Wildlife Management, 8, 223–231.

[ece370255-bib-0136] Tahvanainen, J. , Helle, E. , Julkunen‐Tiitto, R. , & Lavola, A. (1985). Phenolic compounds of willow bark as deterrents against feeding by mountain hare. Oecologia, 65, 319–323. 28310435 10.1007/BF00378905

[ece370255-bib-0137] Taiz, L. , & Zeiger, E. (2006). Secondary metabolites and plant defense. Plant Physiology, 4, 315–344.

[ece370255-bib-0138] Teeling, E. C. , Springer, M. S. , Madsen, O. , Bates, P. , O'Brien, S. J. , & Murphy, W. J. (2005). A molecular phylogeny for bats illuminates biogeography and the fossil record. Science, 307, 580–584. 15681385 10.1126/science.1105113

[ece370255-bib-0139] Ten Brink, H. , Gremer, J. R. , & Kokko, H. (2020). Optimal germination timing in unpredictable environments: The importance of dormancy for both among‐and within‐season variation. Ecology Letters, 23, 620–630. 31994356 10.1111/ele.13461 PMC7079161

[ece370255-bib-0140] Tipton, K. W. , Floyd, E. H. , Marshall, J. G. , & McDevitt, J. B. (1970). Resistance of certain grain sorghum hybrids to bird damage in Louisiana 1. Agronomy Journal, 62, 211–213.

[ece370255-bib-0141] Toole, E. H. , Hendricks, S. B. , Borthwick, H. A. , & Toole, V. K. (1956). Physiology of seed germination. Annual Review of Plant Physiology, 7, 299–324.

[ece370255-bib-0143] Traveset, A. , & Willson, M. F. (1997). Effect of birds and bears on seed germination of fleshy‐fruited plants in temperate rainforests of southeast Alaska. Oikos, 80, 89–95.

[ece370255-bib-0144] Tucker, M. A. , Busana, M. , Huijbregts, M. A. , & Ford, A. T. (2021). Human‐induced reduction in mammalian movements impacts seed dispersal in the tropics. Ecography, 44, 897–906.

[ece370255-bib-0145] Valenta, K. , Brown, K. A. , Rafaliarison, R. R. , Styler, S. A. , Jackson, D. , Lehman, S. M. , Chapman, C. A. , & Melin, A. D. (2015). Sensory integration during foraging: The importance of fruit hardness, colour, and odour to brown lemurs. Behavioral Ecology and Sociobiology, 69, 1855–1865.

[ece370255-bib-0146] Valenta, K. , Nevo, O. , Martel, C. , & Chapman, C. A. (2017). Plant attractants: Integrating insights from pollination and seed dispersal ecology. Evolutionary Ecology, 31, 249–267.

[ece370255-bib-0147] van Leeuwen, C. H. , Soons, M. B. , Vandionant, L. G. , Green, A. J. , & Bakker, E. S. (2023). Seed dispersal by waterbirds: A mechanistic understanding by simulating avian digestion. Ecography, 2023, e06470.

[ece370255-bib-0148] Van Sumere, C. F. (1972). Biochemical studies in relation to the possible germination regulatory role of naturally occurring coumarin and phenolics. Recent Advances in Phytochemistry, 4, 165–221.

[ece370255-bib-0149] Viitala, J. , Korplmäki, E. , Palokangas, P. , & Koivula, M. (1995). Attraction of kestrels to vole scent marks visible in ultraviolet light. Nature, 373, 425–427.

[ece370255-bib-0150] Vorobyev, M. , Osorio, D. , Bennett, A. T. , Marshall, N. J. , & Cuthill, I. C. (1998). Tetrachromacy, oil droplets and bird plumage colours. Journal of Comparative Physiology A, 183, 621–633. 10.1007/s003590050286 9839454

[ece370255-bib-0151] Vuolo, M. M. , Lima, V. S. , & Junior, M. R. M. (2019). Phenolic compounds: Structure, classification, and antioxidant power. In Bioactive compounds (pp. 33–50). Woodhead Publishing.

[ece370255-bib-0152] Waterman, P. G. , & Mole, S. (1994). Analysis of phenolic plant metabolites.

[ece370255-bib-0153] Wheelwright, N. T. , & Janson, C. H. (1985). Colors of fruit displays of bird‐dispersed plants in two tropical forests. The American Naturalist, 126, 777–799.

[ece370255-bib-0154] Wilkie, S. E. , Robinson, P. R. , Cronin, T. W. , Poopalasundaram, S. , Bowmaker, J. K. , & Hunt, D. M. (2000). Spectral tuning of avian violet‐and ultraviolet‐sensitive visual pigments. Biochemistry, 39, 7895–7901. 10891069 10.1021/bi992776m

[ece370255-bib-0155] Willemsen, R. W. , & Rice, E. L. (1972). Mechanism of seed dormancy in *Ambrosia artemisiifolia* . American Journal of Botany, 59, 248–257.

[ece370255-bib-0156] Williams, R. D. , & Hoagland, R. E. (1982). The effects of naturally occurring phenolic compounds on seed germination. Weed Science, 30, 206–212.

[ece370255-bib-0157] Willson, M. F. , Graff, D. A. , & Whelan, C. J. (1990). Color preferences of frugivorous birds in relation to the colors of fleshy fruits. The Condor, 92, 545–555.

[ece370255-bib-0158] Willson, M. F. , & Whelan, C. J. (1989). Ultraviolet reflectance of fruits of vertebrate‐dispersed plants. Oikos, 55, 341–348.

[ece370255-bib-0159] Wilson, G. P. , Evans, A. R. , Corfe, I. J. , Smits, P. D. , Fortelius, M. , & Jernvall, J. (2012). Adaptive radiation of multituberculate mammals before the extinction of dinosaurs. Nature, 483, 457–460. 22419156 10.1038/nature10880

[ece370255-bib-0160] Xiong, F. , Komenda, J. , Kopecký, J. , & Nedbal, L. (1997). Strategies of ultraviolet‐B protection in microscopic algae. Physiologia Plantarum, 100, 378–388.

[ece370255-bib-0161] Xu, G. , Cao, J. , Wang, X. , Chen, Q. , Jin, W. , Li, Z. , & Tian, F. (2019). Evolutionary metabolomics identifies substantial metabolic divergence between maize and its wild ancestor, teosinte. The Plant Cell, 31(9), 1990–2009. 31227559 10.1105/tpc.19.00111 PMC6751114

[ece370255-bib-0162] Yang, Y. , Lin, Y. , & Shi, L. (2021). The effect of lizards on the dispersal and germination of *Capparis spinosa* (*Capparaceae*). PLoS One, 16, e0247585. 33635876 10.1371/journal.pone.0247585 PMC7909692

[ece370255-bib-0163] Yin, H. L. , Li, J. H. , Li, B. , Chen, L. , Li, J. , Tian, Y. , Liu, S. J. , Zhao, Y. K. , Xiao, Y. H. , & Dong, J. X. (2014). Two new coumarins from the seeds of Solanum indicum. Journal of Asian Natural Products Research, 16(2), 153–157. 24152107 10.1080/10286020.2013.841142

[ece370255-bib-0164] Zarei, M. , Azizi, M. , & Bashir‐Sadr, Z. (2011). Evaluation of physicochemical characteristics of pomegranate (*Punica granatum* L.) fruit during ripening. Fruits, 66, 121–129.

[ece370255-bib-0165] Zuntini, A. R. , Carruthers, T. , Maurin, O. , Bailey, P. C. , Leempoel, K. , Brewer, G. E. , Epitawalage, N. , Françoso, E. , Gallego‐Paramo, B. , Ginnie, C. , Negrão, R. , & Knapp, S. (2024). Phylogenomics and the rise of the angiosperms. Nature, 629, 1–8. 10.1038/s41586-024-07324-0 PMC11111409 38658746

